# Higher cut-off values of non-invasive methods might be needed to detect moderate-to-severe steatosis in morbid obese patients: a pilot study

**DOI:** 10.1038/s41598-020-71723-2

**Published:** 2020-09-14

**Authors:** Daniella Braz Parente, Hugo Perazzo, Fernando Fernandes Paiva, Carlos Frederico Ferreira Campos, Carlos José Saboya, Silvia Elaine Pereira, Felipe d’Almeida e Silva, Rosana Souza Rodrigues, Renata de Mello Perez

**Affiliations:** 1 D’Or Institute for Research and Education, Rua Diniz Cordeiro 30, 3º andar, Botafogo, Rio de Janeiro CEP 22281-100 Brazil; 2 Federal University of Rio de Janeiro, Av. Professor Rodolpho Paulo Rocco 255, Cidade Universitária, Ilha Do Fundão, Rio de Janeiro, Rio de Janeiro CEP 21941-913 Brazil; 3 Oswaldo Cruz Foundation - National Institute of Infectious Diseases Evandro Chagas, Av. Brasil, 4365, Manguinhos, Rio de Janeiro, CEP 21040-360 Brazil; 4 Institute of Physics of São Carlos, University of São Paulo, Av. Trabalhador São-Carlense 400, Pq. Arnold Schimidt, São Carlos, São Paulo CEP 13566-590 Brazil; 5 University of the State of Rio de Janeiro, R. S Francisco Xavier, 524, Maracanã, Rio de Janeiro, Rio de Janeiro CEP 20550-013 Brazil; 6 Clinica Cirúrgica Carlos Saboya, Rua Dona Mariana 143/F11, Botafogo, Rio de Janeiro CEP 22280-020 Brazil

**Keywords:** Gastroenterology, Hepatology, Risk factors

## Abstract

To evaluate the diagnostic value of described thresholds of controlled attenuation parameter (CAP) and biomarker scores for liver steatosis and to evaluate new cut-offs to detect moderate-to-severe steatosis (S2–3) in patients with morbid obesity. In this prospective study, 32 patients with morbid obesity with indications for bariatric surgery (15 women and 17 men, mean age = 36 years, median BMI = 40.2 kg/m^2^) underwent CAP, magnetic resonance spectroscopy (MRS), three biomarker scores (Steato-ELSA, Fatty Liver Index (FLI), and Hepatic Steatosis Index (HSI)), and liver biopsy. Subjects were divided into an exploratory cohort (reliable CAP and liver biopsy) and a confirmatory cohort (reliable CAP and MRS) to evaluate new thresholds for CAP and biomarker scores to detect S2–3. Receiver operator characteristic (ROC) curves analyses were performed and the optimal cut-off points were identified using the maximal Youden index. A total of 22 patients had CAP measure and liver biopsy (exploratory cohort) and 24 patients had CAP measure with MRS (confirmatory cohort). New cut-offs were identified for detection of S2–3 by the non-invasive tests using liver biopsy as the reference standard (exploratory cohort). Considering the new proposed cut-offs for detection of S2–3 for CAP (≥ 314 dB/m), Steato-ELSA (≥ 0.832), FLI (≥ 96), and HSI (≥ 53), for the exploratory and confirmatory cohorts sensitivities were: 71–75%, 86–81%, 85–81%, and 71–69% and specificities were: 94–89%, 75–63%, 63–63%, and 75–88%, respectively. Higher cut-offs for CAP and biomarker scores may be better to diagnose moderate-to-severe steatosis in patients with morbid obesity.

## Introduction

Obesity is a major world health problem with increasing prevalence. Patients with morbid obesity, a special part of the obese population, are even more likely to have non-alcoholic fatty liver disease (NAFLD)^1^. The clinical presentation of NAFLD ranges from isolated steatosis to non-alcoholic steatohepatitis (NASH), which may progress to fibrosis, cirrhosis, and hepatocellular carcinoma (HCC). NASH-related HCC may also appear in patients without cirrhosis^2,3^. The severity of the steatosis has been associated with an increased risk of NASH and cirrhosis^4^. Therefore, the correct determination of liver steatosis grade has important implications for prognostic, therapeutic, and monitoring purposes. The prevalence of NAFLD and NASH in patients with morbid obesity may be up to 95% and 56%, respectively^4–6^. Screening is challenging and usual imaging methods have lower accuracy in this group^7^.

Historically, liver biopsy has been considered the reference for detection and grading of liver steatosis^8,9^. However, this invasive method has been challenged by feasibility and sampling error^10,11^. In the last decade, non-invasive tests have been used as an alternative to liver biopsy for NAFLD assessment. Recent international guidelines have reported magnetic resonance spectroscopy (MRS) as the gold-standard for liver fat assessment^7^. Controlled attenuation parameter (CAP) is a relatively new ultrasound method that simultaneously estimates liver steatosis and fibrosis by transient elastography using Fibroscan (EchoSens, France, Paris)^12–16^. In addition, several biomarker scores that combine clinical features, anthropometric measures, and laboratory parameters have been described and validated to detect liver steatosis for NAFLD diagnosis, such as the Fatty Liver Index (FLI)^17^, Hepatic Steatosis Index (HSI)^18^, and Steato-ELSA^19^.

Previous studies reported variable thresholds of CAP to grade liver steatosis using different methods as the reference^12–16^. Although many papers that assess the diagnostic performance of CAP and biomarker scores for detection of moderate-to-severe steatosis in the general population have been published, there is scarce data on the performance of these methods in patients with morbid obesity. The aim of this study was to evaluate CAP and three biomarker scores in patients with morbid obesity to diagnose moderate-to-severe steatosis compared with liver biopsy and MR spectroscopy.

## Methods

### Study population

This cross-sectional study consecutively included adult patients (> 18 years) with clinical indication for bariatric surgery from an outpatient clinic (Rio de Janeiro, Brazil) from December 2013 to November 2017. Inclusion criteria were severe obesity, body mass index (BMI) ≥ 35 kg/m^2^ with at least one comorbidity, or morbid obesity (BMI ≥ 40 kg/m^2^) not responding to medical treatment. Exclusion criteria were abusive alcohol intake (> 20 g/day for women and > 30 g/day for men), long-term use of hepatotoxic drugs, chronic liver disease other than NAFLD/NASH, and clinical or psychological contraindications for bariatric surgery. The study protocol was approved by the Ethics Committee from the D’Or Institute for Research and Education (IRB number 15725613.3.0000.5249). All participants signed an informed consent upon enrollment in the study.

### Clinical evaluation and blood tests

Clinical records included gender, age, body mass index (BMI), waist circumference (WC), type 2 diabetes, and blood hypertension. The following blood tests were performed after an overnight fasting: aspartate transaminase (AST), alanine transaminase (ALT), gamma-glutamyl transferase (GGT), alkaline phospatase, albumin, platelet count, glucose, glycated hemoglobin (HbA1c), insulin, total cholesterol, low-density lipoprotein (LDL)-cholesterol, high-density lipoprotein (HDL)-cholesterol and triglycerides. The homeostasis model assessment of insulin resistance (HOMA-IR) was calculated as HOMA-IR = (fasting glucose (mg/dl)*fasting insulin (µUI/ml))/405^20^. Metabolic syndrome was defined as central obesity plus any two out of four additional factors: hypertriglyceridaemia, reduced HDL-cholesterol, high blood pressure, and raised fasting plasma glucose^21^.

### Controlled attenuation parameter

Controlled attenuation parameter was obtained using the M probe of Fibroscan 502 (EchoSens, Paris, France). All examinations were performed by the same physician (F.D.S., 5 years of experience). Patients fasting for at least 4 h were positioned in dorsal decubitus with right arm extended above the head. The probe was placed perpendicular to the skin at an intercostal space adjacent to the right lobe of the liver. CAP measure was found to be reliable when there were 10 successful measurements, an interquartile range (IQR) lower than 30% of the median value of liver stiffness value, and a success rate greater than 60%. CAP result was calculated as the median of 10 valid measurements.

### Biomarker scores

FLI includes BMI, WC, triglycerides, and GGT; HSI uses aminotransferases, BMI, and the presence of type 2 diabetes adjusted for gender; and Steato-ELSA includes BMI, WC, HOMA-IR, triglycerides, AST, and ALT. These tests were calculated according to the following formulas^17–19^:

$$ {\text{FLI }} = \frac{{\left( {{\text{e}}^{{0.{953}*{\ln}\left( {{\text{triglycerides}},{\text{ mg}}/{\text{dl}}} \right) + 0.{139}*{\text{BMI }} + \, 0.{718}*{\ln}\left( {{\text{GGT}}} \right) \, + \, 0.0{53}*{\ln}\left( {{\text{WC}}} \right) \, - { 15}.{745}}} } \right) \times 100}}{{{1 } + \, \left( {{\text{e}}^{{0.{953}*{\ln}\left( {{\text{triglycerides}},{\text{ mg}}/{\text{dl}}} \right) + 0.{139}*{\text{BMI }} + \, 0.{718}*{\ln}\left( {{\text{GGT}}} \right) \, + \, 0.0{53}*{\ln}\left( {{\text{WC}}} \right) \, - { 15}.{745}}} } \right)}} $$

$$ {\text{HSI }} = { 8 } \times {\text{ ALT}}/{\text{AST ratio }} + {\text{ BMI }} + { 2 }\left( {\text{if type 2 diabetes}} \right) \, + { 2 }\left( {\text{if female}} \right) $$

$$ {\text{Steato}} - {\text{ELSA}} = \frac{{2.71828^{{\left( {\left( {0.0823{\text{*BMI}}} \right) + \left( {0.0337{\text{*WC}}} \right) + \left( {0.0596{\text{*HOMA}} - {\text{IR}}} \right) + \left( {0.0036{\text{*triglycerides~in~mg}}/{\text{dL}}} \right) + \left( {0.0173{\text{*ALT}}} \right) - \left( {0.0124{\text{*AST}}} \right) - 6.6434} \right)}} }}{{1 + 2.71828^{{\left( {\left( {0.0823{\text{*BMI}}} \right) + \left( {0.0337{\text{*WC}}} \right) + \left( {0.0596{\text{*HOMA}} - {\text{IR}}} \right) + \left( {0.0036{\text{*triglycerides~in~mg}}/{\text{dL}}} \right) + \left( {0.0173{\text{*ALT}}} \right) - \left( {0.0124{\text{*AST}}} \right) - 6.6434} \right)}} }} $$

### Magnetic resonance spectroscopy (MRS)

MRS was performed on a 1.5T Philips Achieva (Philips Medical Systems, The Netherlands). A whole-body transmitter coil and a sixteen-element, receive-only, phased-array coil were used. All patients were supine. Patients suspended respiration at the end of inspiration for breath-hold sequences. Axial and coronal T2 images were obtained for anatomical reference. Single-voxel MRS data was acquired from a 25 × 25 × 25 mm voxel obliquely positioned on the Couinaud segment V. Care was taken to avoid liver edges, large hepatic vessels, and the biliary tree. Water suppression was not performed and automated shimming generated water line widths of 40–50 Hz.

Stimulated Echo Acquisition Mode (STEAM) was used for MRS acquisition. For T2 correction, a multi-echo version was used and 5 spectra were collected at echo times (TE) of 15, 25, 35, 45, 55 ms and mixing time of 13 ms. To minimize T1 effects, the repetition time (TR) was 3,500 ms.

#### MRS analysis

MRS data was analyzed by a senior MR physicist (F.F.P., 10 years of experience), blinded to histological diagnosis. Analysis used the Advanced Method for Accurate, Robust, and Efficient Spectral (AMARES) algorithm included in jMRUI. At each echo time, the water (4.7 ppm) and fat (0.5–3 ppm) peaks were measured. Each measurable peak area was individually corrected for T2 decay using nonlinear least-square fits to determine their relative proton density. The relative proton densities of the fat peaks located underneath the water peaks were determined according to Hamilton et al.^22^. The total fat proton density was defined as the sum of all T2-corrected individual fat peaks. The proton density fat fraction (PDFF) was calculated as the ratio of the fat proton density to the sum of the fat and water proton densities. We used the threshold for moderate-to-severe steatosis adapted for patients with morbid obesity defined as MRS PDFF ≥ 6.6%^23^.

### Liver biopsy

Liver biopsy was performed using either a 16-gauge Menghini biopsy needle or by wedge biopsy technique during bariatric surgery. The liver specimens were formalin-fixed, paraffin-embedded and stained with haematoxylin–eosin, Masson’s trichrome, reticulin, and Perls. All slides were read by an experienced pathologist (C.F.F.C.) blinded to patients’ characteristics and laboratory or imaging results. Biopsies were evaluated according to the NASH Clinical Research Network (NASH-CRN) scoring system as normal (< 5%), mild steatosis (5–33%), moderate steatosis (33–66%), or severe steatosis (> 66%) based on the proportion of fat-replete hepatic cells. Diagnosis of NASH was based on the presence of steatosis, hepatocyte injury (ballooning), and lobular inflammation. Fibrosis was staged according to Kleiner & Brunt criteria (stage 0 = none, stage 1 = perisinusoidal or periportal, stage 2 = perisinusoidal and portal/periportal, stage 3 = bridging, and stage 4 = cirrhosis)^9^.

### Statistical analyses

Categorical variables were reported as absolute values (n) and relative frequency (%) and continuous variables as median and interquartile range (IQR). Subjects included in the study were divided into an exploratory cohort (those with reliable CAP and liver biopsy) and a confirmatory cohort (those with reliable CAP and MRS) in order to propose new thresholds to detect moderate-to-severe steatosis using CAP and biomarker scores and to validate them. Receiver operator characteristic (ROC) curves analyses were performed and the optimal cut-off points to detect moderate-to-severe steatosis for CAP, FLI, HSI, and Steato-ELSA were identified using the maximal Youden index. The diagnostic value of the CAP, FLI, HSI, and Steato-ELSA standard cut-offs for the general population (CAP > 268 dB/m^24^, FLI ≥ 96^17^, HSI ≥ 53^18^, Steato-Elsa ≥ 0.832^19^) and the new proposed thresholds were assessed using correct classified (CC) patients, sensitivity (Se), specificity (Sp), positive predictive value (PPV), negative predictive value (NPV), positive (LR+), and negative likelihood ratio (LR−). Areas under the ROC (AUROC) were compared using the empirical non-parametric method according to Delong et al.^25^. Statistical analyses were performed using STATA 15 (StataCorp. 2017. Stata Statistical Software: Release 15. College Station, TX: StataCorp LLC).

### Ethical approval

All procedures performed in studies involving human participants were in accordance with the ethical standards of the institutional and/or national research committee and with the 1964 Helsinki declaration and its later amendments or comparable ethical standards.

### Informed consent

Informed consent was obtained from all individual participants included in the study.

## Results

A total of 47 patients with morbid obesity that underwent bariatric surgery were eligible. Fifteen patients were excluded: 14 due to absence of liver biopsy or MRS and 1 due to an unreliable CAP exam. Therefore, 32 patients with morbid obesity (53% male, median BMI = 40.2 kg/m^2^ (IQR 38.9–42.2), median ALT = 33 UI/L (IQR 20–58), 9% with type-2 diabetes and 47% with blood hypertension) were included. The median CAP value was 308 dB/m (IQR 277–352). The characteristics of patients included in the study are shown in Table 1. A total of 22 patients had CAP measure and liver biopsy (exploratory cohort) and 24 patients had CAP measure with MRS (confirmatory cohort). The median delay between non-invasive assessment and liver biopsy was 2.1 (0.4–3.8) months for the exploratory cohort. In addition, CAP and MRS were performed on the same day as the blood sample for serological markers was collected for patients in the confirmatory cohort. The median distance between skin and liver capsule was 24.8 mm (IQR 22.2–28.2) and only two patients (6%) had a skin-liver capsule distance higher than 35 mm. Most patients from the exploratory cohort had no fibrosis (stage 0), only six patients (28%) had fibrosis stage 1 and no patient had perisinusoidal and portal/periportal fibrosis (stage 2), bridging fibrosis (stage 3) or cirrhosis (stage 4).

**Table 1:** Characteristics of patients with morbid obesity included in the study.

	All (n = 32)
**Clinical and demographic data**	
Male sex	17 (53)
Age, years	36 (30–48)
BMI, kg/m^2^	40.2 (38.9–42.2)
WC, cm	123 (111–132)
Diabetes	3 (9)
Hypertension	15 (47)
**Biochemistry**	
ALT, UI/l	33 (20–58)
AST, UI/l	23 (15–35)
GGT, UI/l	47 (25–73)
Alkaline phosphatases, UI/l	75 (61–93)
Albumin, mg/dl	4.5 (4.2–4.6)
Platelet count, × 10^9^/mm^3^	245 (219–326)
Glucose, mg/dl	95 (86–100)
HbA1c, %	5.6 (5.4–6.0)
Insulin, µUI/ml	22.2 (16.7–30.7)
HOMA-IR	4.98 (3.48–6.32)
Total cholesterol, mg/dl	206 (173–225)
LDL-cholesterol, mg/dl	127 (103–144)
HDL-cholesterol, mg/dl	41 (35–54)
Triglycerides, mg/dl	134 (105–168)
**Controlled attenuation parameter**	
CAP, dB/m	308 (277–352)
IQR, dB/m	23 (15–30)
Success rate, %	100 (91–100)
**Liver biopsy-n = 22**	
< 5%	0 (0)
5–33%	8 (36)
33–66%	8 (36)
> 66%	6 (28)
**MRS-n = 24**	
Steatosis, %	11.9% (6.1–22.6)
< 2.95%	2 (8)
2.95–6.59%	6 (25)
≥ 6.60%	16 (67)
**Biomarker scores for steatosis**	
Steato-ELSA	0.863 (0.797–0.935)
Fatty Liver Index (FLI)	98 (95–99)
Hepatic Steatosis Index (HSI)	53 (49–57)

Data expressed as (a) absolute (%) or (b) median (IQR).

*ALT* alanine transaminase, *AST* aspartate transaminase, *BMI* Body Mass Index, *CAP* controlled attenuation parameter, *HbA1c* glycated hemoglobin, *GGT* gamma-glutamyltransferase, *HDL* high-density lipoprotein, *HOMA-IR* homeostasis model assessment of insulin resistance, *IQR* interquartile range, *LDL* low-density lipoprotein, *MRS* magnetic resonance spectroscopy, *WC* waist circumference.

### Diagnostic accuracy of non-invasive tests in the exploratory cohort

Moderate-to-severe steatosis (≥ 33% of hepatocytes) was present in 64% of patients (95% confidence interval CI 41–82) in the exploratory cohort (n = 22, liver biopsy as the reference). AUROCs (95%CI) of CAP, Steato-ELSA, FLI, and HSI for detection of moderate-to-severe steatosis were 0.897 (0.769–1.000), 0.884 (0.737–1.000), 0.777 (0.576–0.978), and 0.777 (0.539–1.000) (p = 0.10), respectively (Table 2). All patients had Steato-ELSA ≥ 0.386, FLI ≥ 60, and HSI ≥ 36. Median CAP values (IQR) were 271 dB/m (239–289), 323 dB/m (288–358), and 343 dB/m (314–395) for patients with mild, moderate, and severe steatosis in liver biopsy. The standard threshold of CAP ≥ 268 dB/m yielded sensitivity of 93% (95% CI 81–100), specificity of 38% (4–71), PPV of 74% and NPV of 75%.

**Table 2:** Diagnostic value of non-invasive tests for detection of moderate-to-severe steatosis in exploratory (≥ 33%-liver biopsy) and validation (fat fraction ≥ 6.6%-MRS) cohort.

	Exploratory cohort (n = 22)-gold standard = liver biopsy						
	Presence of moderate-to-severe steatosis (≥ 33%) 64% (95% CI 41–82)						
	AUROC (95% CI)						
Controlled attenuation parameter (CAP)	0.897 (0.769–1.000)						
Steato-ELSA	0.884 (0.737–1.000)						
Fatty Liver Index (FLI)	0.777 (0.576–0.978)						
Hepatic Steatosis Index (HSI)	0.777 (0.539–1.000)						
p value	0.1009						

	CC (%)	Sensitivity (95% CI)	Specificity (95% CI)	PPV (%)	NPV (%)	LR+	LR−
**Standard cut-off (Karlas**^24^**)**							
CAP ≥ 268 dB/m	74	93% (81–100)	38% (4–71)	74	75	1.49	0.18
**Proposed cut-offs according to the Maximal Youden Index**							
CAP ≥ 314 dB/m	80	71% (48–95)	94% (78–100)	95	67	12.1	0.30
Steato-ELSA ≥ 0.832	82	86% (67–100)	75% (45–100)	86	75	3.45	0.19
FLI ≥ 96	77	85% (68–100)	63% (29–96)	80	71	2.29	0.23
HSI ≥ 53	73	71% (48–95)	75% (45–100)	83	60	2.86	0.38

	Confirmatory cohort (n = 24)-gold standard = MRS-PDFF						
	Presence of moderate-to-severe steatosis (≥ 6.6%) 67% (95% CI 44–83)						
Controlled attenuation parameter (CAP)	0.813 (0.633–0.992)						
Steato-ELSA	0.805 (0.625–0.984)						
Fatty Liver Index (FLI)	0.836 (0.673–0.999)						
Hepatic Steatosis Index (HSI)	0.813 (0.631–0.994)						
p value	0.9773						

	CC (%)	Sensitivity (95% CI)	Specificity (95% CI)	PPV (%)	NPV (%)	LR+	LR−
**Standard cut-off (Karlas**^24^**)**							
CAP ≥ 268 dB/m	67	88% (71–100)	25% (0–55)	70	50	1.16	0.50
**Proposed cut-offs according to the Maximal Youden Index from the exploratory cohort (liver biopsy as the gold standard)**							
CAP ≥ 314 dB/m	80	75% (54–96)	89% (68–100)	92	67	6.75	0.28
Steato-ELSA ≥ 0.832	75	81% (62–100)	63% (29–96)	81	63	2.17	0.30
FLI ≥ 96	75	81% (62–100)	63% (29–96)	81	63	2.17	0.30
HSI ≥ 53	75	69% (46–92)	88% (65–100)	92	58	5.50	0.36

All patients with morbid obesity had Steato-ELSA > 0.386; FLI > 60 and HSI > 36. Proposed cut-offs for non-invasive tests were based on the maximal Youden Index from the exploratory cohort using liver biopsy as the reference.

^a^Head-to-head comparisons: Steato-ELSA vs CAP (p = 0.06); Steato-ELSA vs FLI (p = 0.05); Steato-ELSA vs HSI (p = 0.19); FLI vs HSI (p = 0.92); FLI vs CAP (p = 0.86); HSI vs CAP (p = 0.80).

### Proposition of new thresholds for detection of moderate-to-severe steatosis

**Figure 1:**
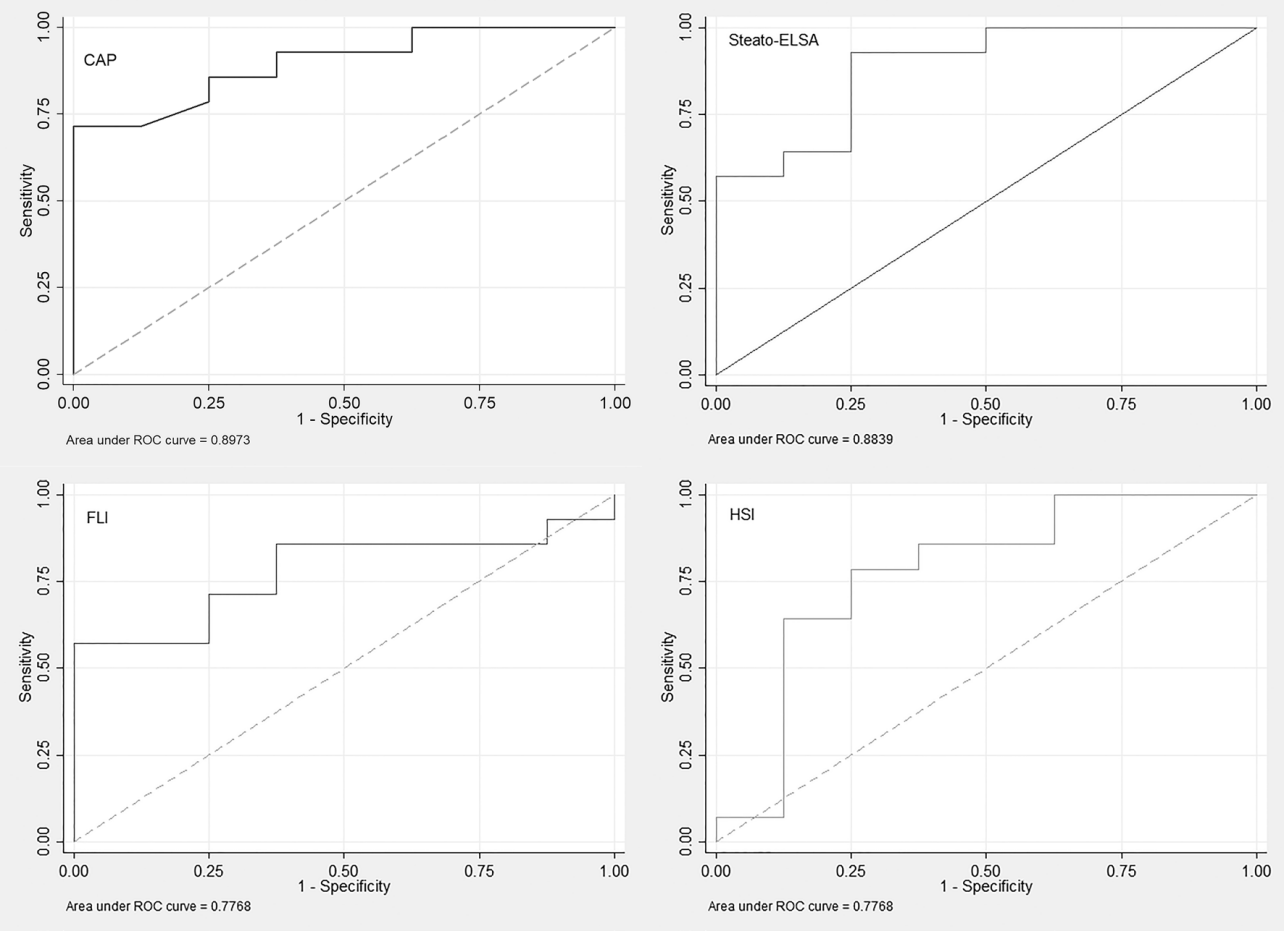
Area under the receiver operator characteristic (AUROC) curves for detection of moderate-to-severe steatosis of Controlled Attenuation Parameter (CAP) (**A**), Steato-ELSA (**B**), Fatty Liver Index (FLI) (**C**), and Hepatic Steatosis Index (HSI) (D) in the exploratory cohort using liver biopsy as the reference.

New cut-offs were proposed for detection of moderate-to-severe steatosis in patients with morbid obesity by the non-invasive tests considering liver biopsy as the reference (exploratory cohort) and the maximal Youden Index (1 − Se + Sp) of each test (Fig. 1). CAP ≥ 314 dB/m yielded sensitivity, specificity, PPV, and NPV of 71% (95% CI 48–95), 94% (95% CI 78–100), 95%, and 67%, respectively. In addition, sensitivities and specificities for Steato-ELSA ≥ 0.832, FLI ≥ 96, and HSI ≥ 53 were Se = 86% (67–100) and Sp = 75% (45–100), Se = 85% (68–100) and Sp = 63% (29–96), Se = 71% (48–95) and Sp = 75% (45–100), respectively (Table 2).

### Diagnostic accuracy of non-invasive test in the confirmatory cohort

**Figure 2:**
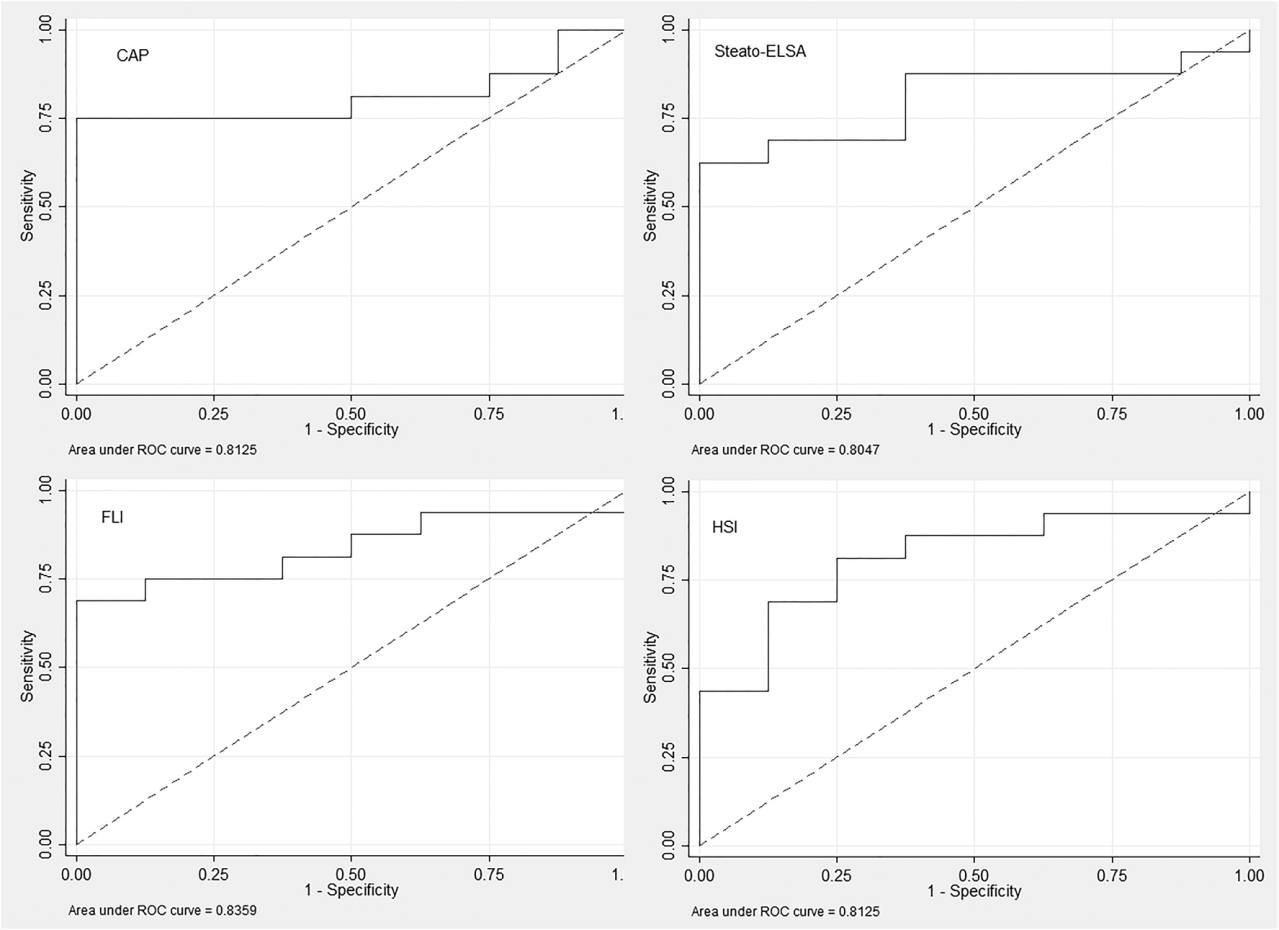
Area under the receiver operator characteristic (AUROC) curves for detection of moderate-to-severe steatosis of Controlled Attenuation Parameter (CAP) (**A**), Steato-ELSA (**B**), Fatty Liver Index (FLI) (**C**), and Hepatic Steatosis Index (HSI) (**D**) in the confirmatory cohort using magnetic resonance spectroscopy (MRS) as the reference.

Moderate-to-severe steatosis in MRS (PDFF ≥ 6.6%) was present in 67% (95% CI 44–83) of patients in the confirmatory cohort (n = 24, MRS as the reference). AUROCs (95% CI) of CAP, Steato-ELSA, FLI, and HSI for detection of moderate-to-severe steatosis were 0.813 (0.633–0.992), 0.805 (0.625–0.984), 0.836 (0.673–0.999) and 0.813 (0.631–0.994) (p = 0.98), respectively (Fig. 2, Table 2). All patients had Steato-ELSA ≥ 0.386, FLI ≥ 60, and HSI ≥ 36. Median CAP values (IQR) were 270 dB/m (255–284), 278 dB/m (274–301) and 350 dB/m (301–368) for patients without steatosis (PDFF < 2.95%), with mild steatosis (PDFF 2.95–6.59%) and with moderate-to-severe steatosis (PDFF ≥ 6.6%) by MRS. The standard threshold of CAP ≥ 268 dB/m yielded sensitivity of 88% (71–100), specificity of 25% (0–55), PPV of 70%, and NPV of 50%. Considering the new proposed cut-offs to detect moderate-to-severe steatosis, sensitivities for CAP (≥ 314 dB/m), Steato-ELSA (≥ 0.832), FLI (≥ 96), and HSI (≥ 53) were 75% (54–96), 81% (62–100), 81% (62–100), and 69% (46–92), respectively. Furthermore, specificity of CAP, Steato-ELSA, FLI, and HSI for diagnosis of moderate-to-severe steatosis were 89% (68–100), 63% (29–96), 63% (29–96), and 88% (65–100). Table 2 summarizes the diagnostic accuracy of non-invasive tests to detect moderate-to-severe steatosis in patients with morbid obesity.

## Discussion

Our study highlighted that classical thresholds of CAP and biomarker scores should be revisited to detect moderate-to-severe liver steatosis in patients with morbid obesity. The present study described high sensitivities but very low specificities considering the general population standard cut-off of CAP ≥ 268 dB/m to detect moderate-to-severe steatosis. Moreover, all patients had FLI ≥ 60, HSI ≥ 36 and Steato-ELSA ≥ 0.386 showing that the standard thresholds of these biomarker scores might be inadequate to these patients. From the results of the maximal Youden index calculations done with liver biopsy as the reference, it can be observed that higher cut-offs should be used to diagnose moderate-to-severe steatosis in patients with morbid obesity. These proposed thresholds were validated in a confirmatory cohort using MRS as the gold standard, which reinforced our hypothesis that non-invasive tests using the general population standard cut-offs led to misclassification of moderate-to-severe steatosis.

A recent meta-analysis using data from the general population with liver biopsy as reference proposed a threshold of CAP ≥ 268 dB/m, and described sensitivity and specificity of 77% and 81%, respectively^24^. However, in our study this threshold yielded high sensitivity (93%) but unacceptably low specificity (38%) for the diagnosis of moderate-to-severe steatosis in patients with morbid obesity. These discordant results might be explained by the difference in the patient’s characteristics between the meta-analysis and our study. The study population of the meta-analysis was composed mostly of patients with chronic viral hepatitis. Only 20% of the sample had NAFLD and patients with morbid obesity were not included. Also, moderate-to-severe steatosis was present in 22% of patients in the meta-analysis compared with 64% in our exploratory cohort. Our population of morbid obese patients in the exploratory cohort had no or minimal fibrosis based on liver biopsy results. This fact was reinforced by the low levels of liver stiffness in both exploratory (5.8 kPa (IQR 5.1–6.3); n = 22) and confirmatory cohorts (5.6 kPa (IQR 4.3–6.7); n = 24). Our study proposed a threshold of CAP ≥ 314 dB/m to detect moderate-to-severe steatosis in patients with morbid obesity based on the maximal Youden index using liver biopsy as the reference. These results suggest that higher thresholds might be needed to grade liver steatosis in patients with morbid obesity. A total of three studies that evaluated the diagnostic value of CAP in patients with morbid obesity proposed cut-offs ranging from 335 to 355 dB/m for the diagnosis of moderate-to-severe steatosis^23,26,27^. Moreover, two studies that included patients with NAFLD and overweight/mild obesity (BMI 27.9 and 30.2 kg/m^2^) described higher cut-offs than the one proposed in the meta-analysis^14,28^. The satisfactory accuracy of the threshold proposed in our study was validated in our confirmatory cohort (sensitivity of 75% and specificity of 89%) that used MRS as the reference.

Similarly, the cut-offs proposed by the original publications of the biomarker scores seem to be inadequate to detect liver steatosis in patients with morbid obesity. In the present study, all patients had biomarker scores higher than the established for the general population (Steato-ELSA ≥ 0.386^19^, Fatty Liver Index (FLI) ≥ 60^17^, and Hepatic Steatosis Index (HSI) ≥ 36^18^). We acknowledge that these biomarker scores were developed to detect liver steatosis (≥ 5%) rather than moderate-to-severe steatosis (≥ 33%). The higher cut-offs proposed in the present study yielded better accuracy for detection of moderate-to-severe steatosis in patients with morbid obesity (Steato-ELSA > 0.832, FLI > 96, and HSI > 53). This was the first study that evaluated the accuracy of Steato-ELSA in patients with morbid obesity, but few studies previously described the diagnostic value of FLI and HSI in this population. The single study that has assessed the diagnostic value of FLI and HSI scores in morbid obese patients proposed higher thresholds to detect moderate-to-severe steatosis in these patients. FLI ≥ 99 showed a sensitivity of 31% and specificity of 80% and HSI ≥ 45 showed a sensitivity of 86% and specificity of 11% to detect moderate-to-severe steatosis^23^.

The major limitation of our study is the relatively small sample size that might hinder the diagnostic accuracy of non-invasive methods to assess moderate-to-severe steatosis. However, morbid obese individuals are a difficult profile of patients to perform liver biopsy and/or MRS. This analysis represents a proof-of-concept that the current thresholds of non-invasive methods might not be adequate to assess moderate-to-severe steatosis in morbid obese patients. Other limitations might be a potential selection bias since we included 32 of 47 eligible patients, and the use of the M probe instead of the XL probe in the analyses. We are aware that CAP measurement using the M probe can be limited by presence of obesity and that the XL probe can be an alternative in these patients. In our study, we chose to evaluate the accuracy of CAP to estimate liver steatosis in morbid obese patients using validated cut-offs from the M probe. Therefore, we have avoided the potential technical bias of using different probes. We acknowledge that a recent study showed lower CAP values using M probe compared with XL probe in the detection of liver steatosis^29^. However, similar accuracy of both probes was observed in these studies despite differences in CAP cut-off values^30,31^. Moreover, despite extremely high BMI (median = 40.2 kg/m^2^), the median distance between skin and liver capsule in the study population was 24.8 mm. In addition, only two patients (6%) had a skin-liver capsule distance higher than 35 mm, in which the use of the XL probe would have been crucial. Lastly, another limitation is the use of wedge and needle liver biopsies. We acknowledge that wedge biopsies may overestimate liver fibrosis staging compared with needle biopsies. However, our study focused on the assessment of liver steatosis and wedge biopsies are as accurate as needle biopsies to assess the degree of steatosis in morbid obese patients^32^. The main strengths of our study rely on the use of two methods considered as the reference for liver steatosis assessment: liver biopsy and MRS. We graded steatosis by MRS using thresholds adapted for patients with morbid obesity^23^. In addition, we used the M probe of FibroScan to assess CAP values due to the extensive validation of this technique compared with the XL probe. Also, a defined cut-off for moderate-to-severe steatosis by meta-analysis is available only for the M probe.

In conclusion, the thresholds of non-invasive tests to detect moderate-to-severe steatosis that were validated in the general population might be inadequate for patients with morbid obesity. This pilot study suggests that higher cut-offs for CAP and biomarker scores might be needed to reduce the rate of steatosis grades misclassification in morbid obese patients. Further studies with a large sample size are recommended to confirm these new thresholds to detect moderate-to-severe steatosis in patients with morbid obesity.
